# Solving third-order boundary value problems with quartic splines

**DOI:** 10.1186/s40064-016-1969-z

**Published:** 2016-03-15

**Authors:** P. K. Pandey

**Affiliations:** Department of Mathematics, Dyal Singh College, University of Delhi, Lodhi Road, New Delhi, 110003 India

**Keywords:** Boundary-value problems, Finite-difference methods, Obstacle problems, Quartic polynomial splines, 65L10, 65L12, 65L20

## Abstract

In this article, we present a novel second order numerical method for solving third order boundary value problems using the quartic polynomial splines. We establish the convergence of the method. We present numerical experiments to demonstrate the efficiency of the method and validity of our second order method, which shows that present method gives better results.

## Background

In this article we consider a quartic splines method for the numerical solution of the third order boundary value problems given as

1 $$ u'''(x)=f(x,u),\quad a\le x \le b , $$

subject to the boundary conditions

$$ u(a)=\alpha , \quad u'(a)=\beta \quad {\text {and}}\quad u'(b)=\gamma $$

where *α*, *β* and *γ* are real constant.

In environments and in most other areas of natural and applied sciences, the differential equations that govern the behavior of model systems are well-known. For instance, to describe the evolution of physical phenomena in fluctuating environments governed by third order differential equation (Ahmad et al. 2012). The study of aero elasticity, sandwich beam analysis and beam deflection theory, electromagnetic waves, theory of thin film flow and incompressible flows and regularization of the Cauchy problem for one-dimensional hyperbolic conservation laws (Bressan 2000) are some other model systems in natural and applied sciences where the third order boundary value problems arise.

The theoretical concepts of existence, uniqueness and convergence of the solution and some specific solution of problem (1) can be found in the literature (Howes 1982; Agarwal 1986; Gupta and Lakshmikantham 1991; Gregus 1987; Murty and Rao 1992; Feckan 1994; Henderson and Prasad 2001; Li 2010). The specific assumption to further ensure existence and uniqueness of the solution to problem (1) will not be considered. Thus the existence and uniqueness of the solution to problem (1) is assumed. Further we assume that problem (1) is well pose. The emphasis in this article will be on the development of an efficient numerical method to deal with approximate numerical solution of the third order boundary value problem.

The quality of a numerical method depends on the accuracy of the method to a great extent. Some efficient and accurate numerical methods for solving higher order boundary value problems are available in literature. Some researchers have studied and solved in particular third order boundary value problems with different boundary conditions using different methods for instance some literary work in Finite Difference Method (Al-Said 2001), Quintic Splines (Khan and Aziz 2003), Non polynomial spline method (Islam et al. 2005, 2007; Srivastava and Kumar 2012), Quartic B-splines (Gao and Chi 2006), Haar wavelets method (Fazal-i-Haq and Ali 2011), Collocation quantic spline (Noor and Khalifa 1994), Reproducing Kernel Method (Li and Wu 2012) and references therein can be found. With advent of computers it gained important to develop more accurate numerical methods to solve higher order boundary value problems. Hence, the purpose of this article is to develop an efficient numerical method for solution of third order boundary value problems (1).

We present our work in this article as follows. In the next section we derive a finite difference method. In section “Convergence analysis”, we discuss convergence of the proposed method under appropriate condition. The application of the proposed method on the test problems and illustrative numerical results so produced to show the efficiency in section “Numerical results”. Discussion and conclusion on the performance of the proposed method present in section “Conclusion”.

## The difference method 

We define *N* finite numbers of nodal points of the domain [*a*, *b*], in which the solution of the problem (1) is desired, as $a\le x_0<x_1<x_2<\cdots <x_N =b$ using uniform step length *h* such that $x_i=a+i.h, i=0,1,2,\ldots ,N$. Also, we let *u*(*x*) be the exact solution of (1) and we denote the numerical approximation of *u*(*x*) at node $x=x_i$ as *u*_*i*_. Let us denote *f*_*i*_ as the approximation of the theoretical value of the source function *f*(*x*, *u*(*x*)) at node $x=x_i ,\; i=0,1,2,\ldots ,N$. Thus the boundary value problem (1) at node $x=x_i$ may be written as

2 $$ u'''_i=f_i ,\quad a\le x_i \le b , $$

subject to the boundary conditions

$$ u_0=\alpha , \quad u'_0=\beta \quad {\text {and}}\quad u'_N=\gamma $$

We wish to determine the numerical approximation of the theoretical solution *u*(*x*) of the problem (1) at the nodal point $x_i , i=1,2,\ldots ,N$.

Define $x_{i-\frac{1}{2}}=x_i-\frac{1}{2}h, i=1,2,\ldots ,N$ nodes in [*a*, *b*]. Let $s_{i-\frac{1}{2}}$ be an approximation to $u_{i-\frac{1}{2}}= u(x_{i-\frac{1}{2}})$ solution of the problem (1) at these nodes obtained by the quartic spline $S_i(x)$ passing through the points $(x_{i-\frac{1}{2}},s_{i-\frac{1}{2}})$ and $(x_{i+\frac{1}{2}},s_{i+\frac{1}{2}})$. For each *i*th segment, we write polynomial quartic spline $S_i(x)$ in the form

3 $$ \begin{aligned}S_i(x)&=c_{i0}\left( x-x_{i-\frac{1}{2}}\right) ^4+c_{i1} \left( x-x_{i-\frac{1}{2}}\right) ^3  +c_{i2}\left( x-x_{i-\frac{1}{2}}\right) ^2\\&\quad+c_{i3} \left( x-x_{i-\frac{1}{2}}\right) +c_{i4} \end{aligned}$$

where $c_{i0} , c_{i1} , c_{i2} , c_{i3} , c_{i4}$ are real finite constants. Then the quartic spline defined by

4 $$ s(x)=S_i(x),\quad i=1,2,\ldots ,N-1 \quad {\text {and}}\quad s(x)\in C^3[a,b]. $$

To determine constants *c*_*i*0_, *c*_*i*1_, *c*_*i*2_, *c*_*i*3_, *c*_*i*4_ we assume that $S_i(x)$ satisfies problem (1) with boundary conditions at $x_{i-\frac{1}{2}}$ and $x_{i+\frac{1}{2}}$ . Following the idea in Fazal-i-Haq and Ali (2011) we let

5 $$\begin{aligned} S_i\left( x_{i-\frac{1}{2}}\right)&=  s_{i-\frac{1}{2}},\quad S_i\left( x_{i+\frac{1}{2}}\right) =s_{i+\frac{1}{2}},\quad S'_i\left( x_{i-\frac{1}{2}}\right) =s'_{i-\frac{1}{2}},  \\ S''\left( x_{i-\frac{1}{2}}\right)&= s''_{i-\frac{1}{2}}\quad {\text {and}}\quad S'''\left( x_{i-\frac{1}{2}}\right) =T_{i-\frac{1}{2}},\quad i=1,2,\ldots ,N-1. \end{aligned}$$

Thus we will obtain

6 $$\begin{aligned} c_{i0}&= \frac{1}{24h}\left( T_{i+\frac{1}{2}}-T_{i-\frac{1}{2}}\right) ,\quad c_{i1}=\frac{1}{6}T_{i-\frac{1}{2}},\quad c_{i2}=-\frac{h}{24}\left( T_{i+\frac{1}{2}}+3T_{i-\frac{1}{2}}\right) ,\\ c_{i3}&=  \frac{1}{h}\left( s_{i+\frac{1}{2}}-s_{i-\frac{1}{2}}\right) ,\quad c_{i4}=s_{i-\frac{1}{2}}, \quad i=1,2,\ldots ,N-1. \end{aligned}$$

Using method of undetermined coefficients and Taylor’s series expansion, we discretize problem (2) at these nodes in [*a*, *b*],

7 $$\begin{aligned} & 9s_{i-\frac{1}{2}}-s_{i+\frac{1}{2}}=  8s_{i-1}+3hs'_{i-1} -\frac{3h^3}{8}T_{i-\frac{1}{2}}+t_i,\quad i=1 \\ &-15s_{i-\frac{3}{2}}+10s_{i-\frac{1}{2}}-3s_{i+\frac{1}{2}}= -8s_{i-2}-\frac{h^3}{48} \left( 165T_{i-\frac{1}{2}}-45T_{i+\frac{1}{2}}\right) +t_i,\quad i=2 \\ & s_{i-\frac{5}{2}}-3s_{i-\frac{3}{2}}+3s_{i-\frac{1}{2}}-s_{i+\frac{1}{2}}= -\frac{h^3}{2}\left( T_{i-\frac{3}{2}}+T_{i-\frac{1}{2}}\right) +t_i,\quad 3\le i \le {N-1} \\ &s_{i-\frac{5}{2}}-3s_{i-\frac{3}{2}}+2s_{i-\frac{1}{2}}= hs'_i+\frac{h^3}{48}\left( -25T_{i-\frac{3}{2}}+21T_{i-\frac{1}{2}}\right) +t_i,\quad i=N \end{aligned}$$

where $t_i, i=1,2,\ldots ,N$ is truncation error. In discretization we have used boundary conditions in a natural way.

After neglecting the *t*_*i*_ in (7), at nodal points $x_{i-\frac{1}{2}}, i=1,2,\ldots ,N$, we will obtain the *N* × *N* linear or nonlinear system of equations depends on the source function *f*(*x*, *u*) in unknown $s_{i-\frac{1}{2}}$. We have to solve a system of equations by an appropriate method. We have applied either Gauss Seidel or Newton–Raphson iterative method to solve above system of Eq. (7) respectively for linear and nonlinear system of equations by following algorithms,

$${\mathbf{S}} ^{(k+1)}=({\mathbf{L}} +{\mathbf{d}} )^{-1}\cdot \left( -{\mathbf{U}} \cdot {\mathbf{S}} ^{k}+{\mathbf{b}} \right) , $$

in which terms are defined as follow,

$$\begin{aligned} {\mathbf{S}} ^{(k+1)}&=  \left( \begin{array}{c} {\text {s}}^{(k+1)}_\frac{1}{2} \\ {\text {s}}^{(k+1)}_\frac{3}{2} \\ \cdots \\ \cdots \\ {\text {s}}^{(k+1)}_{N-\frac{1}{2}} \\ \end{array} \right) _{N\times 1} , \quad {\mathbf{L}} +{\mathbf{d}} =\left( \begin{array}{c c c c c c c } 9 &\quad & \quad &\quad &\quad &\quad &\quad 0 \\ -15 &\quad 10 &\quad & \quad &\quad &\quad &\quad \\ 1 &\quad -3 &\quad 3 &\quad &\quad &\quad &\quad \\ &\quad 1 &\quad -3 &\quad 3 &\quad &\quad &\quad \\ \cdots &\quad \cdots &\quad \cdots &\quad \cdots &\quad \cdots &\quad \cdots &\quad \cdots \\ \cdots &\quad \cdots &\quad \cdots &\quad \cdots &\quad \cdots &\quad \cdots &\quad \cdots \\ & \quad &\quad &\quad 1 &\quad -3 &\quad 3 &\quad \\ 0 &\quad &\quad &\quad &\quad 1 &\quad -3 &\quad 2 \\ \end{array} \right) _{N\times N} \\ {\mathbf{U}}= &  \left( \begin{array}{c c c c c c c } 0&\quad -1 &\quad &\quad &\quad &\quad &\quad 0 \\ &\quad 0&\quad -3 &\quad &\quad &\quad &\quad \\ &\quad &\quad 0 &\quad -1 &\quad &\quad &\quad \\ &\quad &\quad &\quad 0 &\quad -1 &\quad &\quad \\ \cdots &\quad \cdots &\quad \cdots &\quad \cdots &\quad \cdots &\quad \cdots &\quad \cdots \\ \cdots &\quad \cdots &\quad \cdots &\quad \cdots &\quad \cdots &\quad \cdots &\quad \cdots \\ &\quad & \quad & \quad & \quad & \quad &\quad -1 \\ 0 &\quad &\quad &\quad &\quad &\quad &\quad 0 \\ \end{array} \right) _{N\times N} , \quad {\mathbf{b}} = (b_i)_{N\times 1} \end{aligned}$$

where

$$\begin{aligned} b_i={\left\{ \begin{array}{ll} 8\alpha +3h\beta -\frac{3h^3}{8}T_{i-\frac{1}{2}}, & \quad i=1 \\ -8\alpha -\frac{5h^3}{16}\left( 11T_{i-\frac{1}{2}}-3T_{i+\frac{1}{2}}\right) , & \quad i=2 \\ -\frac{h^3}{2}\left( T_{i-\frac{3}{2}}+T_{i-\frac{1}{2}}\right) , & \quad 3\le i\le N-1 \\ h\gamma +\frac{h^3}{48}\left( -25T_{i-\frac{3}{2}}+21T_{i-\frac{1}{2}}\right) , & \quad i=N \end{array}\right. } \end{aligned}$$

and

$$ F_i\left( s_\frac{1}{2},s_\frac{3}{2},\ldots ,s_{N-\frac{1}{2})}\right) =0 ,\quad i=1,2,\ldots ,N. $$

Let **s** and **F** are the all vector of values $s_{i-\frac{1}{2}}$ and *F*_*i*_ respectively.

$${\mathbf{F}} ({\mathbf{s}} +\delta {\mathbf{s}} )= {\mathbf{F}} ({\mathbf{s}} )+{\mathbf{J}} \delta {\mathbf{s}} +O\left( (\delta {\mathbf{s}} )^2\right) .$$

Neglecting the term $O((\delta {\mathbf{s}} )^2)$ and assuming ${\mathbf{s}} +\delta {\mathbf{s}}$ is root of **F**, then

$${\mathbf{s}} _{m+1}={\mathbf{s}} _m+\delta {\mathbf{s}} ,\quad {\text {and}} \quad \delta {\mathbf{s}} =-{\mathbf{J}} ^{-1}{} {\mathbf{F}} , \quad m=0,1,2\ldots . $$

where ${\mathbf{J}} ^{-1}=\frac{\partial (f_1,f_2,\ldots ,f_N)}{\partial (s_\frac{1}{2},s_\frac{3}{2},\ldots ,s_{N-\frac{1}{2}})}$. Also some time we consider    $\delta {\mathbf{s}} ={\mathbf{s}} _{m+1}-{\mathbf{s}} _m$.

We compute numerical value of *u*_*N*_ by using following second order approximation,

8 $$ u_N=s_{N-\frac{1}{2}}+\frac{1}{2}hs'_N. $$

## Convergence analysis

We will consider following linear test equation for convergence analysis of the proposed method (7).

9 $$ u'''{(x)}= f(x) , \quad a\le x\le b . $$

subject to the boundary conditions $u_0=\alpha , \quad u'_0=\beta$ and $u'_N=\gamma$. We can write the proposed method (7) in the matrix form as

10 $$ {\mathbf{Du}} ={\mathbf{a}} +{\mathbf{t}} $$

where

$$\begin{aligned} {\mathbf{D}} =\left( \begin{array}{c c c c c c c } 9 &\quad -1 &\quad &\quad &\quad &\quad & \quad 0 \\ -15 &\quad 10 &\quad -3 &\quad & \quad & \quad &\quad \\ 1 &\quad -3 &\quad 3 &\quad -1 &\quad &\quad &\quad \\ &\quad 1 &\quad -3 &\quad 3 &\quad -1 &\quad &\quad \\ \cdots &\quad \cdots &\quad \cdots &\quad \cdots &\quad \cdots &\quad \cdots &\quad \cdots \\ \cdots & \quad \cdots &\quad \cdots &\quad \cdots &\quad \cdots &\quad \cdots &\quad \cdots \\ &\quad &\quad &\quad 1 &\quad -3 &\quad 3 &\quad -1 \\ 0 &\quad &\quad &\quad &\quad 1 &\quad -3 &\quad 2 \\ \end{array} \right) _{N\times N} \end{aligned}$$

and ${\mathbf{u}} =(u_{i-\frac{1}{2}}), {\mathbf{a}} =(a_i)$, and ${\mathbf{t}} =(t_i)$ are *N*-dimensional column vectors defined as,

$$\begin{aligned} a_i&= \left\{ \begin{array}{ll} 8\alpha +3h\beta -\frac{3h^3}{8}T_{i-\frac{1}{2}}, &\quad i=1 \\ -8\alpha -\frac{5h^3}{16}\left( 11T_{i-\frac{1}{2}}-3T_{i+\frac{1}{2}}\right) , & \quad i=2 \\ -\frac{h^3}{2}\left( T_{i-\frac{3}{2}}+T_{i-\frac{1}{2}}\right) , & \quad 3\le i\le N-1 \\ h\gamma +\frac{h^3}{48}\left( -25T_{i-\frac{3}{2}}+21T_{i-\frac{1}{2}}\right) , & \quad i=N \end{array} \right. \\ t_i&= \left\{ \begin{array}{ll} -\frac{27h^5}{1920}u^{(5)}_{i-\frac{1}{2}}, & \quad i=1\\ -\frac{7h^5}{8}u^{(5)}_{i-\frac{1}{2}}, & \quad i=2 \\ o(h^6), & \quad 3\le i\le N-1 \\ \frac{31h^5}{1920}u^{(5)}_{i-\frac{1}{2}}, & \quad i=N\\ \end{array}\right. \end{aligned}$$

Let us solve test problem (9) by proposed method (7) after neglecting the terms $t_i$. We will obtain a system of linear equations in $s_{i-\frac{1}{2}}$. Solving the system of equations so obtained by an iterative method, we get an approximate solution. Let us write system of equation in matrix form,

11 $${\mathbf{Ds}} ={\mathbf{a}} $$

where ${\mathbf{s}} =(s_{i-\frac{1}{2}})$ is *N*-dimensional column vector of approximate solution of system of equations obtained from (7). Let us define

12 $$ e_{i-\frac{1}{2}}=u_{i-\frac{1}{2}}-s_{i-\frac{1}{2}} $$

where $s_{i-\frac{1}{2}}$ is an approximate value of $u_{i-\frac{1}{2}}, i=1,2,\ldots ,N$. Thus from (10) and (11) we can write an error equation

13 $${\mathbf{De}} ={\mathbf{t}} $$

where ${\mathbf{e}} =(e_{i-\frac{1}{2}}), i=1,2,\ldots ,N$ is *N*-dimensional column vector. Let ${\mathbf{K}} =(k_{ij})$ be the explicit inverse of nonsymmetric Toeplitz matrix **D** and defined as Jain et al. (1987), Varga (2000), Horn and Johnson (1990), [24],

14 $$k_{ij}={\left\{ \begin{array}{ll} \frac{(2i-1)(4N-2i+1)}{24N}, & \quad 1\le i \le N, \quad j=1 \\ (2i-1)^2c_1, & \quad i \le j \le N,\quad 2\le j \\ \frac{(N-i)(N-i+1)}{2}c_2-\frac{(N-i+2)(N-i-1)}{2}c_3,& \quad j+1 \le i \le N-1 \end{array}\right. } $$

where

15 $$ c_1= {\left\{ \begin{array}{ll} \frac{(N+1-j)(2j+1)^2}{40N\left( 8j-(2j-5)^2\right) }, & \quad j=2 \\ \frac{N+1-j}{8N}, & \quad 2< j\le N \end{array}\right. } $$

16 $$ c_2= {\left\{ \begin{array}{ll} \frac{(N+1-j)(4N(j-1)+1)-8(j-1)}{24N}, & \quad j=2 \\ \frac{(N+1-j)(4N(j-1)+1)-8(j-1)}{8N},& \quad j+1\le i< N \end{array}\right. } $$

17 $$ c_3=  {\left\{ \begin{array}{ll} \frac{(N+1-j)(4N(j-1)+1)}{24N}, & \quad j=2 \\ \frac{(N+1-j)(4N(j-1)+1)}{8N}, & \quad j+1\le i< N \end{array}\right. } $$

From (15), (16) and (17), we can prove that **K** is nonsymmetric and positive matrix. Let matrix ${\mathbf{R}} =(R_{i1})_{N\times 1}$, denotes the matrix of the row sum of the matrix ${\mathbf{K}} =(k_{ij})_{N\times N}$ where,

18 $$ R_{i1}=\sum ^{N}_{j= 1} k_{ij} $$

Hence we have obtained

19 $$\Vert {\mathbf{K}} \Vert =\max _{\begin{array}{c} 1\le i\le N \end{array}}\left| R_{i1}\right| =\frac{4N^4-(9N^2-9N-2)}{48N} $$

Thus for large *N*, from (19) we conclude that

20 $$ \Vert {\mathbf{K}} \Vert \le \frac{(b-a)^3}{12h^3} $$

Let

21 $$ M= \max _{\begin{array}{c} x \end{array}\in [a, b]} \left| u^{(5)}(x)\right| , $$

Then from (13), (20) and (21) we have

22 $$ \Vert {\mathbf{e}} \Vert \le \frac{7h^2(b-a)^3}{96}M $$

Thus from Eq. (22) it follows that $\Vert {\mathbf{e}} \Vert \rightarrow 0$ as $h \rightarrow 0$. This establishes the convergence of the method (7) and the order of convergence of method (7) is at least $O(h^2)$.

## Numerical results

To illustrate our method and demonstrate its computational efficiency, we have considered three model problems. In each model problem, we took uniform step size *h*. In Tables 1, 2, 3 and 4, we have shown *MAE* the maximum absolute error in the solution *u* of the problems (1) for different values of *N*. We have used the following formula in computation of *MAE*,

$$ MAE= max {\left\{ \begin{array}{ll} {\left| u(x_i)-s(x_i)\right| } & \quad 1\le i \le {N-1} \\ {\left| u(x_i)-u_i\right| } & \quad i =N \end{array}\right. } $$

We have used Gauss Seidel and Newton–Raphson iteration method to solve respectively for linear and nonlinear system of equations arised from Eq. (7). All computations were performed on a Windows 2007 Ultimate operating system in the GNU FORTRAN environment version 99 compiler (2.95 of gcc) on Intel Core i3-2330M, 2.20 Ghz PC. The solutions are computed on *N* nodes and iteration is continued until either the maximum difference between two successive iterates is less than 10^(−10)^ or the number of iteration reached 10^3^.

**Problem 1** The model linear problem given by

$$ u'''(x)=u(x)+f(x),\quad 0\le x\le 1 $$

subject to boundary conditions

$$ u(0)=0, \quad u'(0)=1 \quad {\text {and}}\quad u'(1)=0 $$

where *f*(*x*) is calculated so that the analytical solution of the problem is $u(x)=x\exp (-x)$. The *MAE* computed by method (7) for different values of *N* are presented in Table 1 and the results obtained in the numerical experiment is compared with high order finite difference method reported in Al-Said (2001).

**Problem 2** The nonlinear model problem given by

$$ u'''(x)=x^4u(x)-u^2(x)+f(x),\quad 0\le x\le 1 $$

subject to boundary conditions

$$ u(0)=0 ,\quad u'(0)=-1\qquad {\text {and}}\quad u'(1)=\sin (1) $$

where *f*(*x*) is calculated so that the analytical solution of the problem is $u(x)=(x-1)\sin (x)$. The *MAE* computed by method (7) for different values of *N* are presented in Table 2 and the results obtained in the numerical experiment is compared with high order finite difference method reported in Al-Said (2001).

**Problem 3** Consider the following third-order obstacle problems (Noor and Khalifa 1994),

$$ u'''(x)={\left\{ \begin{array}{ll} 0, & \quad 0\le x \le \frac{1}{4} \\ u(x)-1, & \quad \frac{1}{4}\le x \le \frac{3}{4}\\ 0, & \quad \frac{3}{4}\le x \le 1 \end{array}\right. } $$

subject to boundary conditions

$$ u(0)=0 \quad ,\quad u'(0)=0 \quad {\text {and}}\quad u'(1)=0 $$

The analytical solution of the problem is

$$ u(x)={\left\{ \begin{array}{ll} \frac{1}{2}a_1x^2, & \quad 0\le x \le \frac{1}{4} \\ 1+ a_2\exp (x)+\exp \left( \frac{-x}{2}\right) \left( a_3\cos \left( \frac{\surd 3}{2}x\right) +a_4\sin \left( \frac{\surd 3}{2}x\right) \right) , & \quad \frac{1}{4}\le x \le \frac{3}{4} \\ \frac{1}{2}a_5x(x-2)+a_6, & \quad \frac{3}{4}\le x \le 1 \end{array}\right. } $$

where the constants $a_i, i=1,2,\ldots ,6$ can be determined by the solving a system of linear equations which can be obtained by applying the continuity conditions of $u(x), u'(x)$ and $u''(x)$ at $x=\frac{1}{4}$ and $\frac{3}{4}$. The *MAEI*, *MAEM* and *MAEE* respectively in interval $[0,\frac{1}{4}], [\frac{1}{4},\frac{3}{4}]$ and $[\frac{3}{4},1]$ and *MAE* = max{*MAEI*, *MAEM*, *MAEE*} computed by method (7) for different values of *N* are presented in Table 3 and the results obtained in the numerical experiment is compared with some higher order method reported in literature presented in Table 4.

**Table 1:** Maximum absolute error (Problem 1)

Method	Maximum absolute error			
	*N* = 4	*N* = 8	*N* = 16	*N* = 32
Equation (7)	.16645713 (−1)	.41207962 (−2)	.10233842 (−2)	.23465362 (−3)
Al-Said (2001)	.10738984 (−1)	.32454655 (−2)	.88991225 (−3)	.23343042 (−3)

**Table 2:** Maximum absolute error (Problem 2)

Method	Maximum absolute error			
	*N* = 8	*N* = 16	*N* = 32	*N* = 64
Equation (7)	.11921225 (−1)	.33391770 (−2)	.87742222 (−3)	.23732412 (−3)
Al-Said (2001)	.16499877 (−1)	.41241050 (−2)	.10325760 (−2)	.27042627 (−3)

**Table 3:** Maximum absolute error (Problem 3)

N	Maximum absolute error			
	*MAEI*	*MAEM*	*MAEE*	*MAE*
8	.74443180 (−3)	.24691788 (−1)	.56693217 (−1)	.56693217 (−1)
16	.11904127 (−4)	.11839149 (−3)	.42345375 (−4)	.11839149 (−3)
32	.29750796 (−4)	.28597564 (−4)	.19483268 (−4)	.29750796 (−4)
64	.74037612 (−5)	.58218491 (−4)	.74401498 (−4)	.74401498 (−4)

**Table 4:** Maximum absolute error (Problem 3)

Method	Maximum absolute error		
	*N* = 16	*N* = 32	*N* = 64
Equation (7)	.119 (−3)	.297 (−4)	.744 (−4)
Al-Said (2001)	.196 (−3)	.489 (−4)	.122 (−4)
Islam et al. (2005)	.712 (−3)	.405 (−3)	.222 (−3)
Gao and Chi (2006)	.113 (−2)	.530 (−3)	.252 (−3)
Noor and Al-Said (2004)	.115 (−2)	.532 (−3)	.256 (−3)
Li and Wu (2012)	.118 (−2)	.547 (−3)	.262 (−3)
Al-Said and Noor (2003)	.123 (−2)	.553 (−3)	.261 (−3)
Noor and Khalifa (1994)	.126 (−2)	.560 (−3)	.310 (−3)
Al-Said et al. (1996)	.689 (−2)	.711 (−2)	.727 (−3)

We have described a numerical method for numerical solution of third order boundary value problem and three model problems including an obstacle problem considered to test the performance of the proposed method. Numerical result for examples for different values of *N* which is presented in tables, the maximum absolute errors in solution decreases with decrease in step size *h*. Also from the numerical results in Table 4, is clear that the new method (7) outperforms the existing methods. On the other hand, it is evident that method (7) is convergent and the rate of convergence is at least quadratic.

## Conclusion

A finite difference method to find the numerical solution of third order boundary value problems has been developed. At nodal point $x = x_{i-\frac{1}{2}}, i = 1, 2,\ldots ,N$ we have obtained a system of algebraic equations given by (7). Thus we have a system of linear equations if source function *f*(*x*, *u*) is linear otherwise system of nonlinear equations. The propose method produces good approximate numerical value of the solution for model problems and it is computationally efficient and accurate method. The idea presented in this article leads to the possibility to develop finite difference methods for the numerical solution of higher odd order boundary value problems. Works in these directions are in progress.
